# Improved plan quality with automated radiotherapy planning for whole brain with hippocampus sparing: a comparison to the RTOG 0933 trial

**DOI:** 10.1186/s13014-017-0896-7

**Published:** 2017-10-02

**Authors:** J. Krayenbuehl, M. Di Martino, M. Guckenberger, N. Andratschke

**Affiliations:** Department of Radiation Oncology, University Hospital Zurich, Rämistrasse 100, CH-8091 Zurich, Switzerland

**Keywords:** Volumetric modulated arc therapy, Automated planning optimization, Whole brain irradiation, Hippocampus sparing, RTOG 0933

## Abstract

**Background:**

Whole-brain radiation therapy (WBRT) with hippocampus sparing (HS) has been investigated by the radiation oncology working group (RTOG) 0933 trial for patients with multiple brain metastases. They showed a decrease of adverse neurocognitive effects with HS WBRT compared to WBRT alone. With the development of automated treatment planning system (aTPS) in the last years, a standardization of the plan quality at a high level was achieved. The goal of this study was to evaluate the feasibility of using an aTPS for the treatment of HS WBRT and see if the RTOG 0933 dose constraints could be achieved and improved.

**Methods:**

Ten consecutive patients treated with HS WBRT were enrolled in this study. 10 × 3 Gy was prescribed according to the RTOG 0933 protocol to 92% of the target volume (whole-brain excluding the hippocampus expanded by 5 mm in 3-dimensions). In contrast to RTOG 0933, the maximum allowed point dose to normal brain was significantly lowered and restricted to 36.5 Gy. All patients were planned with volumetric modulated arc therapy (VMAT) technique using four arcs. Plans were optimized using Auto-Planning (AP) (Philips Radiation Oncology Systems) with one single AP template and optimization.

**Results:**

All the constraints from the RTOG 0933 trial were achieved. A significant improvement for the maximal dose to 2% of the brain with a reduction of 4 Gy was achieved (33.5 Gy vs. RTOG 37.5 Gy) and the minimum hippocampus dose was reduced by 10% (8.1 Gy vs. RTOG 9 Gy). A steep dose gradient around the hippocampus was achieved with a mean dose of 27.3 Gy at a distance between 0.5 cm and 1 cm from the hippocampus. The effective working time to optimize a plan was kept below 6′.

**Conclusion:**

Automated treatment planning for HS WBRT was able to fulfil all the recommendations from the RTOG 0933 study while significantly improving dose homogeneity and decreasing unnecessary hot spot in the normal brain. With this approach, a standardization of plan quality was achieved and the effective time required for plan optimization was minimized.

## Background

Whole-brain radiation therapy (WBRT) was for decades the standard treatment for patient with multiple brain metastases [1] by various malignancies or for prophylactic cranial irradiation for patients with small cell lung carcinoma. However, the use of WBRT has decreased in the past years due to reports on possible equivalence of an SRS only approach, the advent of targeted therapies with CNS activity [2, 3] and the recognition of adverse neurocognitive effects from WBRT which are believed to be caused from the damage to the hippocampus due to irradiation [4]. With the improvement of radiation therapy (RT) delivery such as intensity modulated radio-therapy (IMRT) or volumetric modulated radiotherapy (VMAT), the concept of hippocampal sparing WBRT has been proposed and investigated with the hypothesis to avoid some of the observed neurocognitive toxicity of conventional WBRT [1].

The hippocampus sparring (HS) is aimed at, but a sufficient dose is required to the remaining brain tissue to avoid the risk of marginal failure in the HS dose gradient. Reassuringly, several reports have shown that the risk of metastases occurring in the hippocampal area is low [5]. Currently, HS-WBRT is also evaluated using in addition a simultaneous integrated boost to increase the probability of local control in macroscopic disease [6, 7].

Recently, HS WBRT for brain metastases has been evaluated in a prospective phase II study (RTOG 0933) [8]. The primary objective was the assessment of the delayed recall by the Hopkins Verbal Learning Test-Revised 4 (HVLT-R) [8]. The results were positive showing no decline in HVLT-R at two and four months and no change in respect to the quality of life in comparison to the historical control group (WBRT) in patients still alive at 6 months [9]. Strict constraints to the target coverage as well as the dose to the organs at risk (OAR) were defined in the radiation oncology working group (RTOG) 0933 protocol. Nevertheless, to achieve a reasonable dose gradient to spare the hippocampus, high doses to the PTV, i.e. the normal brain, were allowed: not more than 2% of the PTV was recommended to receive a cumulative dose of 37.5 Gy and 40 Gy was still allowed. Automated treatment planning system (aTPS) have gained attention, as they may allow an automation of the optimization process leading to a standardization of the plan quality at a high level, surpassing in some cases the manually optimized plans [10–12].

The goal of this study was to evaluate the feasibility of using an automated treatment planning system (aTPS) for the treatment of HS WBRT to achieve a) consistent planer-independent plan quality and b) to significantly reduce the high dose to normal brain while still achieving all RTOG 0933 dose constraints.

## Methods and materials

### Automatic VMAT optimization

The automated treatment planning module, Auto-Planning (AP), included in Pinnacle 14.0 (Philips Radiation Oncology Systems, Fitchburg, WI) was used for plan optimization. It is a fully integrated module in the TPS, similar to the “manual” inverse optimizer module and has been previously described [10, 13]. Briefly, Pinnacle AP is a template-knowledge based treatment planning system. During AP, the optimizer automatically runs multiple times with the individual optimization goals, constraints and weights automatically added and adjusts the priority of clinical goals based on their probability of being achieved. A collapsed cone convolution algorithm was used to calculate the dose distribution (version 14.0).

### Ethics approval and patient selection

All patients included in this study have given their approval to use their data for scientific research. For this treatment planning study, ten consecutive patients treated for HS WBRT were enrolled.

### Structure definition

Computer tomography and magnetic resonance imaging were registered and fused. These images were used for the delineation of the hippocampus, eyes, lens, lacrimal glands and whole brain. The planning target volume (PTV) was defined as the whole-brain expanded by 5 mm in three dimensions excluding the hippocampal avoidance regions as recommended in the RTOG 0933 protocol. The hippocampal avoidance regions were defined as the hippocampal expanded by 5 mm in three dimensions.

### VMAT optimization using an automated treatment planning system

The plan optimization was automated using AP where the clinical objectives and priorities for each PTV and OAR were defined. The model created in Auto-Planning was optimized based on two cases to achieve or improve all recommended constraints from RTOG 0933 protocol, see Table 1. These two cases were not included in the set of patients used for the plan evaluation.

**Table 1:** Dose volume histogram, monitor units and treatment time for hippocampus sparing whole brain irradiation

	HS WBRT				
	Krayenbuehl et al. Mean (range)	RTOG 0933 recommendation	Gondi et al. Mean (range)	Nevelsky et al. Mean (range)	Wang et al. Mean (range)
PTV D98% (Gy)	25.8 (25.0–27.1)	≥ 25Gy	N/A	25.7 (25.4–25.9)	
PTV V95% (%)	96.4 (95.2–97.8)	N/A	96.9 (96.1–97.5)	N/A	96.9 (96.0–97.5)
PTV D2 (Gy)	33.5 (32.8–34.6)	≤37.5	N/A	37.2 (36.9–37.6)	35.1 (34.8–35.6)
PTV Dmax (Gy)	36 (35.1–36.5)	N/A	N/A	N/A	N/A
PTV V30Gy (%)	92 (92.0–92.0)	= 90	N/A	92.1 (90.5–93.2)	N/A
PTV HI	0.24 (0.21–0.26)	N/A	0.3 (0.26–0.34)	0.36 (0.34–0.37)	0.26 (0.23–0.33)
PTV Dmean (Gy)	31.5 (30.9–32.2)	N/A	N/A	N/A	N/A
HC D100% (Gy)	8.1 (7.8–8.5)	≤ 9	N/A	8.4 (7.7–8.9)	9.3 (8.3–10.0)
HC Dmean (Gy_2_)	7.3 (6.0–7.9)	N/A	7.3 (7.2–7.6)	N/A	N/A
HC Dmax (Gy)	14.1 (12.0–15.3)	≤ 16	15.3 (14.3–15.9)	14.3 (13.5–15.4)	16 (14.6–16.9)
Lens Dmax (Gy)	4.6 (3.7–5.6)	N/A	3.8 (3.1–4.3)	N/A	5.8 (4.5–6.5)
Opt chiasm Dmax (Gy)	32.9 (31.7–35.1)	≤ 37.5	N/A	36.2 (33.9–37.2)	34.7 (33.1–36.8)
Opt nerve Dmax (Gy)	33.1 (32.5–33.8)	≤ 37.5	N/A	32.5 (28.3–35.7)	32.0 (23.7–36.1)
Lacrymal glands Dmax (Gy)	10.8 (6.6–15.5)	≤ 37.5	N/A	N/A	N/A
MU	1481 (1345–1550)	N/A	N/A	1724 (1622–1914)	N/A
Effective working time (min)	4.5 (4.1–5.3)	N/A	N/A	N/A	N/A

*Abbreviations*: *PTV*: planning target volume, *D98%*: dose to 98% of the volume, *V95%*: volume covered by 95% of the prescribed dose, *D2%*: dose to 2% of the volume, *Dmax*: maximal point dose, *V30Gy*: volume covered by 95% of the prescribed dose, *HI*: homogeneity index, *HC*: hippocampus, *MU*: monitor units, Gy2: equivalent dose if the actual treatment was delivered in 2 Gy per fraction with an α/β ratio of 2 Gy, *MU*: monitor units

All patients were planned with a VMAT technique using four arcs: two 360° co-planar arcs, and two non-coplanar arcs with couch at 300° and 60° with arcs ranging from 181° to 10° moving clockwise and 350° to 179° moving clockwise. The plans were optimized for a Trilogy Linac (Varian Medical System, Palo Alto, CA) with a 60 pair multileaf collimator (MLC) of 5 mm width (Millennium MLC).

One treatment plan was generated for each patient using AP and only one optimization cycle was performed. The plan parameters used for the optimization are listed in Table 2. For optimization purposes, a target help structure (PTV_7mm), defined as the PTV minus the hippocampus expanded by 7 mm in three-dimension in order to allow the optimizer to fulfil all the objectives. This structure was used only for optimization purposes, see Table 2, and not for the plan normalization. This was the only help structure generated for the optimization step. 10 × 3 Gy was prescribed to 92% of the PTV.

**Table 2:** Dose constraints for plan optimization and tuning balance

Structure	DVH parameter	Dose (Gy)	Priority
PTV_7mm	–	30	–
Hippocampus	Max dose	14.5	High
	D40%	10	Medium
	D99%	8	High
Lens	Max dose	5	High
Lacrimal gland	Max dose	20	Medium
	Mean dose	10	Medium

Tuning balance

Conform to Target: 30%

Dose fall-off Margin: 2.6 cm

Hot-Spot Maximum goal: 105%

*Abbreviations*: *PTV_7mm* planning target volume minus the hippocampus expanded by 7 mm in three-dimension, *D99%* dose to 99% of the volume, *D40%* dose to 40% of the volume

Quality assurance for each of the ten clinical AP plans was performed on a phantom (Delta4, ScandiDos AB, Uppsala, Sweden). All fields had to have a gamma value >95% with a distance to agreement of 3 mm and a dose difference of 3%.

### Plan evaluation

Dose–volume histograms (DVH) were calculated for the PTVs and OARs of each plan. All plans were normalized to 92% of the PTV volume (V92%) receiving 30 Gy.

Plan results were compared to published DVH parameters for HS WBRT [1, 14, 15]. For the PTV, dose to 2% (D2%) and 98% (D98%), homogeneity index (HI) defined as (D2%-D98%)/Dmedian, and percent of the target volume receiving 95% of the prescribed dose (V95%) were evaluated. The hippocampus dose was evaluated based on its minimal dose (D100%), maximal dose (Dmax) and mean 2Gy equivalent dose calculated with an α/β ratio of 2 Gy. The other OARs, lens, lacrimal glands, optic chiasm and optical nerve were evaluated based on the maximal point dose.

**Fig. 1:**
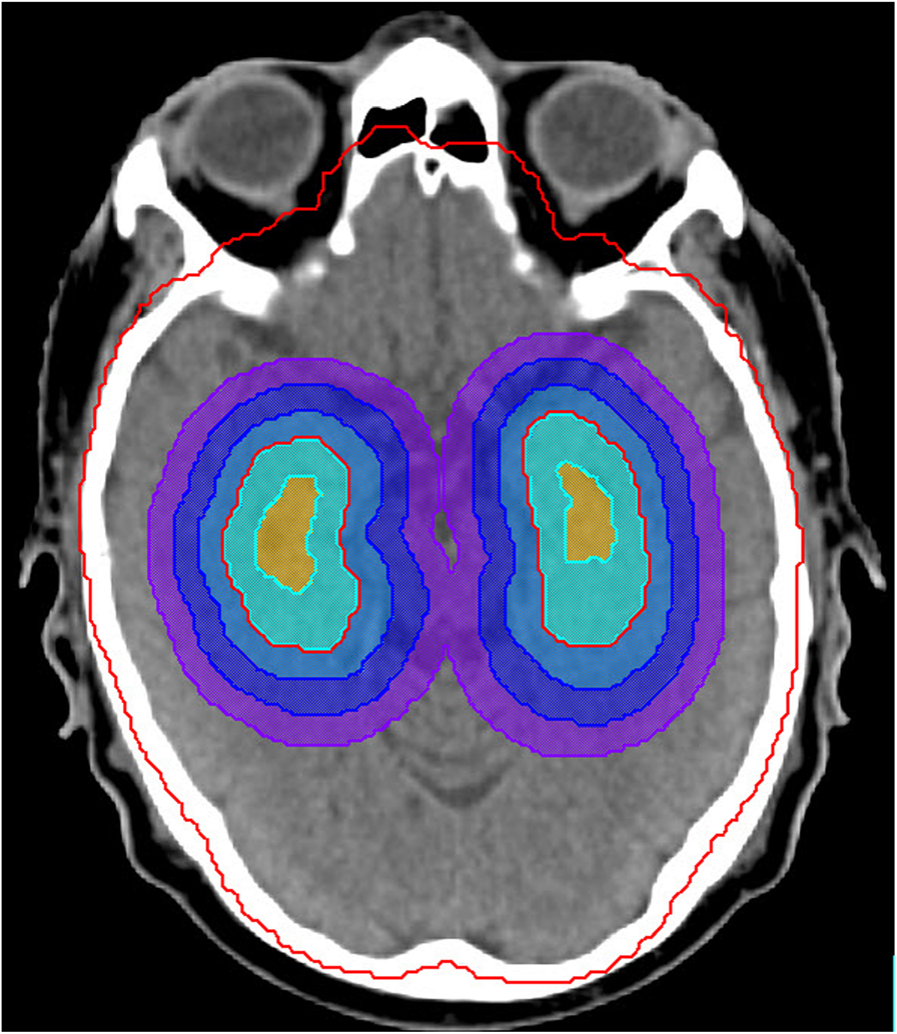
Ring structures around the hippocampus. The hippocampus is segmented in orange, the target in red and the first, second, third and fourth ring structures in light blue, blue, dark blue and violet respectively. All the ring structures have a width of 5 mm

Four ring structures around the hippocampus were generated in order to evaluate the dose gradient between the hippocampus and the target. The first ring was generated by applying a margin of 5 mm to the hippocampus in 3-dimensions. The second, third and fourth ring was defined as an outer margin of 5 mm in 3-dimensions to the first, second and third ring respectively, see Fig. 1. The minimal, maximal and mean dose to these ring structures were evaluated.

In addition to the DVH parameter evaluation, the planning time and the number of monitor units (MU) were reported and evaluated. The planning time was defined as the effective working time starting when the target and OAR volumes are defined by the clinicians and finishing when the plans were optimized by a dosimetrist.

## Results

Target and OAR objectives and priorities used for the optimization of HS WBRT with AP are listed in Table 2. These settings were used for the optimization of all cases planned with Auto-Planning. No individual optimization was performed. The plans were normalized to 92% of the target volume receiving 30Gy. The target was defined as the brain subtracted by the hippocampal expanded by 5 mm in three-dimension.

### Target volumes

D2% and D98% of the target volume were 33.1 Gy ranging from 32.6 Gy to 33.6 Gy and 25.8 Gy ranging from 25.0 Gy to 27.8 Gy respectively, fulfilling and even surpassing significantly the RTOG 0933 constraints. The target mean dose was 31.5 Gy ranging from 30.9 Gy to 32.2 Gy. The homogeneity index was 0.24 ranging from 0.21 to 0.26. The volume covered by 95% of the prescribed dose was larger than 95.2% for every patient with a mean value of 96.4%. The maximal point for every plan was kept below 36.5 Gy.

**Fig. 2:**
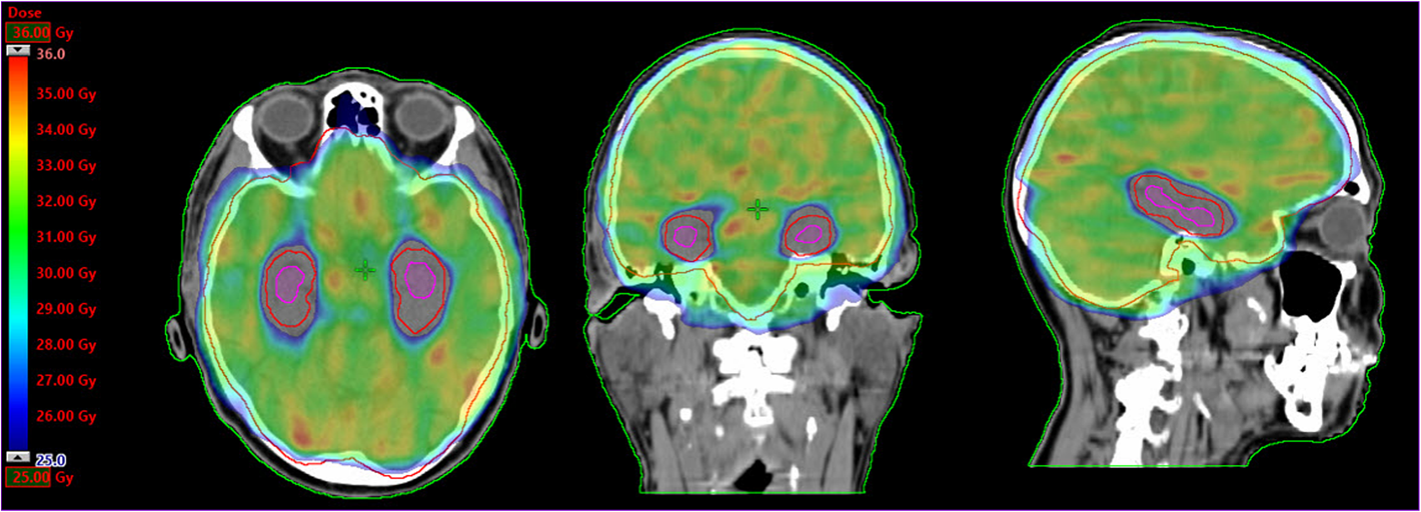
Dose in close proximity to the hippocampus. Abbreviations: *Dmin*: Minimal dose, *Dmean*: Mean dose, *Dmax*: Maximal dose

The dose in the ring structures around the hippocampus are displayed in Fig. 2. The mean dose within 5 mm to the hippocampus was 16.8 Gy with a minimal dose of 8.8 Gy and a maximal dose of 27.2 Gy. The mean dose increased to 27.3 Gy, 31.0 Gy and 31.7Gy in the second third and fourth ring respectively.

### Organs at risk

The OAR doses are summarized in Table 1. AP was able to achieve all of the RTOG 0933 constraints. The mean D100% to the hippocampus was 8.1 Gy ranging from 7.8 Gy to 8.5 Gy and the mean 2Gy equivalent dose was 7.3 Gy ranging from 6.0 Gy to 7.6 Gy.

The mean maximal dose to the optical nerve was 33.1 Gy ranging from 32.5Gy to 33.8 Gy and the maximal dose for the lacrimal glands was always kept bellow 15.5 Gy, far below the recommended of 37.5 Gy dose from the RTOG 0933. The maximal dose to the optic chiasm was kept below 35.1 Gy for every cases, below the recommendation of 37.5Gy from RTOG protocol.

### Planning time and monitor units

The working planning time, defined as the effective working time to generate a plan was kept below 6 min for each plan optimized. The average time was 4.5’ ranging from 4.1’ to 5.3’. The mean MU was 1481 MU ranging from 1448 MU to 1554 MU.

**Fig. 3:**
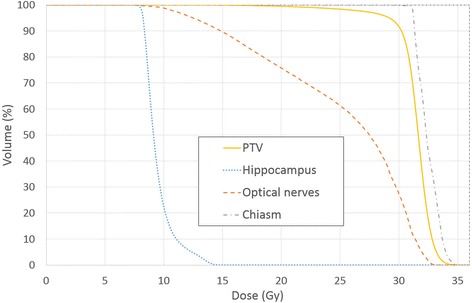
Average dose volume histogram over ten patients planned with HS WBRT

**Fig. 4:**
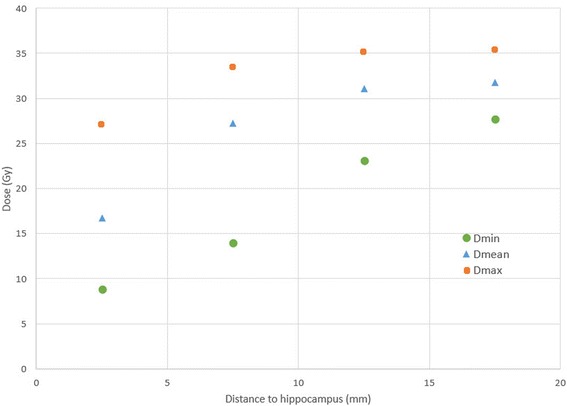
Dose distribution in axial, coronal and sagittal view for a patient planned with HS WBRT with 10 × 3 Gy

The DVH averaged over the ten cases planned with HS WBRT are plotted in Fig. 3. The DVH parameters, monitor units and effective working time to optimize a HS WBRT plan are summarized in Table 1. A typical dose distribution for HS WBRT in axial, coronal and sagittal view are displayed on Fig. 4.

## Discussion

HS WBRT has gained much attention as an attractive concept and RTOG 0933 was a significant achievement to show a clinical relevant benefit of this approach. However, plan optimization of HS WBRT is a complex process, where hard constraints have to be fulfilled. A compromise between minimal dose to the hippocampus, target coverage and maximal dose to the target needs to be achieved. Multiple iterations are normally required before all the constraints are fulfilled. After each iteration, the planner has to modify the constraints, generate new help structures to take into account the cold and hot spots until a satisfactory dose distribution is achieved.

Our hypothesis was that with an automated planning approach a) all these complex planning steps could be automated resulting in a reduction of the effective working time [10], b) a reproducible high quality dose distribution can be achieved independent of the planner experience and c) volumes of normal brain receiving hotspots higher than 115% can be avoided.

The effective working time for plan optimization for automated planning of HS WBRT was in the order of five minutes. Beside the reduction of working time required to generate a plan for HS WBRT, an improvement of the average plan quality was achieved. Indeed, all the RTOG constraints were easily fulfilled after a single optimization. The two main improvements in the plan quality were a reduction of the hippocampus minimal dose by 10% and a reduction of the D2% for the target from 125 to 112% without compromising PTV coverage required within RTOG 0933. We consider especially the latter an achievement, i.e. significantly less high dose within the normal brain (2% normal brain receiving 33.5Gy compared to 37.2 Gy and 37.5 Gy (8, 14) the most relevant finding for clinical practice.

In the study performed by Gondi et al., for HS WBRT, using linac-based IMRT fields, 30Gy was prescribed in ten fractions [1]. They reported a maximum dose of 15.3 Gy and a medium dose of 7.8 Gy to the hippocampus. D2% to the brain was not reported but dose larger than 40 Gy (133% of the prescribed dose) were displayed on the DVH, see Table 1. The plan optimization was done with nine non-coplanar IMRT fields using seven different couch angles. With such a beam arrangement, one can assume that the treatment delivery would be time consuming.

In the study performed by Nevelsky et al. using a linac-based IMRT nine fields, only two different couch angles were used in order to reduce the treatment delivery time [14]. They were able to reduce the maximal dose to the hippocampus compared to Gondi et al. to 14.3 Gy, see Table 1. A mean minimal dose of 8.4 Gy to the hippocampus was reported. These values are very similar to our results were a value of 14.1 Gy and 8.1 Gy for maximal and minimal dose were obtained. The D2% to the brain achieved by Nelesky et al. was 37.2 Gy (124% of the prescribed dose), close to the maximal recommended value from the RTOG protocol of 37.5 Gy and an HI of 0.36 was achieved. In our study the D2% and the HI were both significantly improved. This led to an improvement of the dose homogeneity and a reduction of hot spot in the brain.

In order to reduce further the treatment delivery, Wang et al. [15] evaluated an automated treatment planning system using two coplanar arcs. Eight of the ten patients had a minimal and maximal hippocampus dose above the recommended value from the RTOG 0933 protocol. There D2% to the PTV was 35.1Gy, below the 37.5Gy recommended values from the protocol. In the current study, four arcs were used which allowed to decrease furthermore the D2% by 1.6Gy and fulfil all the recommended constraints from the RTOG 0933 protocol. The increase of arcs will have an impact on the treatment delivery time.

In the RTOG 0933, 98% of the target volume should receive at least 25Gy in order to avoid cold spot in the brain which could lead to an increase of the local relapse (LR). The brain region receiving the lowest dose is in close proximity to the hippocampus avoidance region. By under-dosing this region, an increase of LR compared to WBRT alone could occur [5]. The LR could increase up to 4% for small-cell lung cancer (SCLC). Therefore, a special attention was taken when choosing the optimization parameters to achieve a high dose gradient around the hippocampus. At a distance from 0.5 cm to 1 cm from the hippocampus, i.e. in the first 5 mm from the hippocampus avoidance region, the mean dose was 27.3 Gy, close to the prescribed dose of 30 Gy.

However, to arrive at an acceptable hippocampus sparing, the PTV constraints developed by Gondi et al. were incorporated into RTOG 0933 and this allowed significant hotspots within the normal brain: D2% was set to 40 Gy allowing normal brain to have hotspots up to 133% of the prescribed dose. Although restricting hippocampal dose has been proven to protect neurocognitive function early, i.e. 4 months, after HS WBRT, its benefits at later time points are not yet known. In contrast, several reports have shown cortical changes like cortical thinning and microvascular perfusion changes after radiotherapy which may counteract initial protective effects on hippocampal function and may negatively affect neurocognitive functions other than those mediated by proper hippocampal function [16, 17].

## Conclusion

With automated treatment planning, a standardization of the plan quality was achieved and the effective time required for planning optimization was kept close to five minutes. aTPS for HS WBRT was able to fulfil all the recommendations from the RTOG 0933 study. Furthermore, the dose to most of the organs at risk could be significantly reduced, there was an improvement in dose homogeneity and a highly clinical relevant decrease of hot spots within the normal brain could be achieved.

## Abbreviations

AP: Auto-Planning
aTPS: Automated treatment planning system
Dmax: Maximal dose
Dmean: Mean dose
Dmin: Minimal dose
DVH: Dose–volume histograms
Dx%: Dose to x% of the volume
HI: Homogeneity index
HS: Hippocampus sparing
HVLT-R: Hopkins Verbal Learning Test-Revised
IMRT: Intensity modulated radiotherapy
LR: Local relapse
MLC: Multileaf collimator
MU: Monitor units
OAR: Organs at risk
PTV: Planning target volume
PTV_7mm: Planning target volume minus the hippocampus expanded by 7 mm in three-dimension
RT: Radiation therapy
RTOG: Radiation oncology working group
VMAT: Volumetric modulated arc therapy
Vx%: Volume receiving x% of the prescribed dose
WBRT: Whole-brain radiation therapy
