# Correction to: Improved plan quality with automated radiotherapy planning for whole brain with hippocampus sparing: a comparison to the RTOG 0933 trial

**DOI:** 10.1186/s13014-017-0901-1

**Published:** 2017-11-08

**Authors:** J. Krayenbuehl, M. Di Martino, M. Guckenberger, N. Andratschke

**Affiliations:** Department of Radiation Oncology, University Hospital Zurich, Rämistrasse 100, CH-8091 Zurich, Switzerland

## Correction

In the original publication [1] figure legends 2 and 4 have been swapped (Additional file 1 and Additional file 2). The original article was updated to rectify this error. The corrected figures are included to this Erratum.

Figure 2 Dose in close proximity to the hippocampus. Abbreviations: *Dmin*: Minimal dose, *Dmean*: Mean dose, *Dmax*: Maximal dose

Figure 4 Dose distribution in axial, coronal and sagittal view for a patient planned with HS WBRT with 10 × 3 Gy

## Additional files

Additional file 1: Dose in close proximity to the hippocampus. Abbreviations: *Dmin*: minimal dose, *Dmean*: mean dose, *Dmax*: maximal dose. (JPG 35 kb)

Additional file 2: Dose distribution in axial, coronal and sagittal view for a patient planned with HS WBRT with 10 x 3 Gy. (146 kb)
