# Supplementary figures and images for: Correction to: Improved plan quality with automated radiotherapy planning for whole brain with hippocampus sparing: a comparison to the RTOG 0933 trial
Source: Radiat Oncol. 2017 Nov 8;12:173. doi: 10.1186/s13014-017-0901-1 (PMC5679153; doi:10.1186/s13014-017-0901-1)

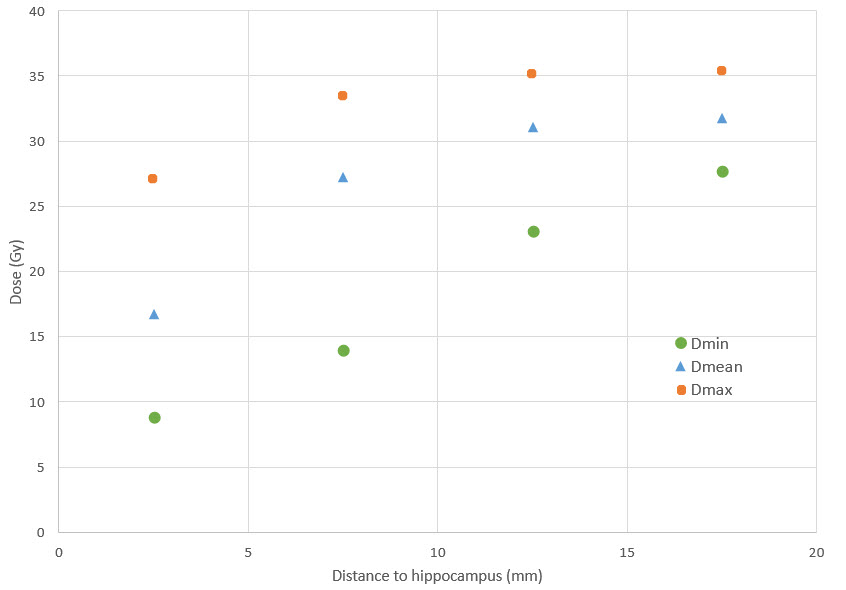

Supplement: Supplementary file 1 — Dose in close proximity to the hippocampus. Abbreviations: Dmin: minimal dose, Dmean: mean dose, Dmax: maximal dose. (JPG 35 kb) [file 13014_2017_901_MOESM1_ESM.jpg]

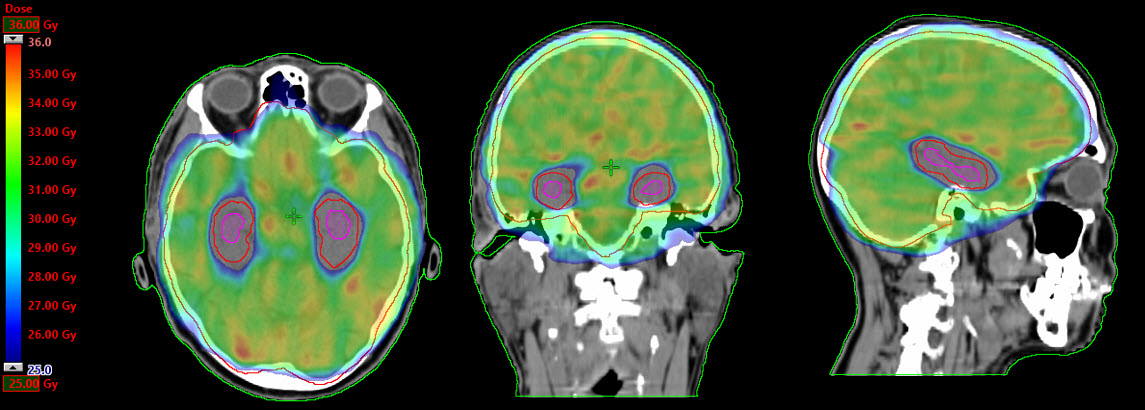

Supplement: Supplementary file 2 — Dose distribution in axial, coronal and sagittal view for a patient planned with HS WBRT with 10 x 3 Gy. (146 kb) [file 13014_2017_901_MOESM2_ESM.jpg]
